# Stress Induces Release of Extracellular Vesicles by *Trypanosoma cruzi* Trypomastigotes

**DOI:** 10.1155/2021/2939693

**Published:** 2021-09-23

**Authors:** Camilla Ioshida Vasconcelos, A Cronemberger-Andrade, Normanda Souza-Melo, Juliana Terzi Maricato, Patrícia Xander, Wagner Luiz Batista, Rodrigo Pedro Soares, Sergio Schenkman, Ana Claudia Torrecilhas

**Affiliations:** ^1^Departamento de Ciências Farmacêuticas, UNIFESP, Rua São Nicolau, 210, 09913-030, Diadema, São Paulo, Brazil; ^2^Cell Therapy Institute, Spinal Cord Injury and Tissue Regeneration Center Salzburg (SCI-TReCS), Paracelsus Medical University (PMU), 5020 Salzburg, Austria; ^3^Departamento de Microbiologia, Imunologia e Parasitologia, UNIFESP, Rua Pedro de Toledo, 669, 04039-032 São Paulo, Brazil; ^4^Departamento de Microbiologia, Imunologia e Parasitologia, UNIFESP, Rua Botucatu, 862, 04023-062 São Paulo, Brazil; ^5^Instituto René Rachou/FIOCRUZ-MG, Av. Augusto de Lima, 1715, 30190-009 Belo Horizonte, Minas Gerais, Brazil

## Abstract

All extracellular forms of *Trypanosoma cruzi*, the causative agent of Chagas disease, release extracellular vesicles (EVs) containing major surface molecules of the parasite. EV release depends on several mechanisms (internal and external). However, most of the environmental conditions affecting this phenomenon are still unknown. In this work, we evaluated EV release under different stress conditions and their ability to be internalized by the parasites. In addition, we investigated whether the release conditions would affect their immunomodulatory properties in preactivated bone marrow-derived macrophages (BMDM). Sodium azide and methyl-cyclo-*β*-dextrin (CDB) reduced EV release, indicating that this phenomenon relies on membrane organization. EV release was increased at low temperatures (4°C) and acidic conditions (pH 5.0). Under this pH, trypomastigotes differentiated into amastigotes. EVs are rapidly liberated and reabsorbed by the trypomastigotes in a concentration-dependent manner. Nitrosative stress caused by sodium nitrite in acid medium or *S*-nitrosoglutathione also stimulated the secretion of EVs. EVs released under all stress conditions also maintained their proinflammatory activity and increased the expression of iNOS, Arg 1, IL-12, and IL-23 genes in IFN-*γ* and LPS preactivated BMDM. In conclusion, our results suggest a budding mechanism of release, dependent on the membrane structure and parasite integrity. Stress conditions did not affect functional properties of EVs during interaction with host cells. EV release variations under stress conditions may be a physiological response against environmental changes.

## 1. Introduction

The flagellated protozoan *Trypanosoma cruzi* is the etiological agent of Chagas disease, affecting 8 million people worldwide. Approximately 100 million people are at risk of infection, causing about 2,000 deaths per year. These circumstances make this disease a serious health problem [1, 2]. In the bloodstream of the mammalian vertebrate host, trypomastigote forms invade various cell types, differentiate into amastigotes, and proliferate in their cytoplasm.

Trypomastigotes derived from infected mammalian cell cultures release extracellular vesicles (EVs) in the culture medium. They express major protozoan surface molecules [2], including mucin-like glycoproteins, glycosylphosphatidyl inositol phospholipids (GIPLs), and members of the gp85/*trans*-sialidase (TS) superfamily of glycoproteins [3]. EV release promotes parasite infectivity and modulates the host's innate and acquired immune responses [4–10]. However, mechanisms involved in EV release by the parasites facing adverse environmental conditions are still poorly understood. Those may include diverse body fluids (e.g., blood), extracellular matrix, and contact with chemical agents. As a part of a wider study on *T. cruzi* EVs, we sought to characterize their release under different conditions (temperature, pH, and chemical agents). We also evaluated if *T. cruzi* EVs released under stress conditions were functionally affected during interaction with bone marrow-derived macrophages.

## 2. Material and Methods

### 2.1. Animal Ethics

The experimental procedures used in this study were approved by the Animal Use Ethics Committee (CEUA) of the Federal University of São Paulo (http://www.unifesp.br/reitoria/ceua) under protocol # 9254110216.

### 2.2. Parasite Cultures

Trypomastigotes (Y-strain) were obtained from the supernatant of infected LLC-MK_2_ cells maintained in low-glucose DMEM (Vitrocell Embriolife), supplemented with 10% fetal bovine serum (SFB) (Vitrocell Embriolife). Trypomastigotes were collected from the cell culture medium from the fifth to the ninth day after infection by centrifugation (1,000 × *g* for 15 min). They were washed with PBS, and the pellet containing the parasites was resuspended in 1 mL DMEM. Parasite concentration was estimated using a Neubauer (Reichert) chamber.

### 2.3. EV Release Assays

Trypomastigotes (1 × 10^7^) were incubated for 2 h in DMEM with/without FBS containing 5% glucose, at distinct temperatures and pH levels, and/or in the presence of metabolic inhibitors and nitroxidative compounds (NaN_3_ and NaNO_2_) and methyl-*β*cyclodextrin (CBD). The assays were always performed in triplicate. After incubation, trypomastigotes were centrifuged (1,000 × *g* for 15 min) (MiniSpin plus, Eppendorf) and supernatants were collected for measuring EVs' size and concentration. To determine viability, 90 *μ*L of the parasite's suspension was added to each well of a 96-well plate and mixed with 10 *μ*L of the PrestoBlue (Thermo Fisher Scientific). Plates were incubated (37°C, 2 h), and fluorescence was detected by excitation at 560 nm and emission at 590 nm (Synergy HT, BioTek). Parasites incubated without any agent were used as negative controls, and the results were expressed as relative fluorescence units. In parallel, trypomastigotes were centrifuged (3,000 × *g*, 5 min) and the supernatant was discarded. The pellet was resuspended in PBS, dried on glass coverslips, fixed in methanol, and washed with 2X running water. Then, parasites were stained with Giemsa (Merck) diluted 1 : 10 in running water and mounted with Entellan (Merck). Images were acquired under an optical microscope. 300 parasites/slide were counted, and *T. cruzi* morphology was analyzed (Imager.A2, Zeiss).

### 2.4. Isolation of T. cruzi Trypomastigote EVs

The total shed material released by the trypomastigotes was centrifuged (10,000 × *g*, 15 min). After centrifugation, the supernatant was filtered using a 0.22 *μ*m filter (Sarstedt) and ultracentrifuged (100,000 × *g*, 1 h, 4°C) (Sorvall WX Ultra Series 80, rotor T890, Thermo Scientific). The pellet containing EVs was resuspended in 5 mL of sterile PBS and submitted to a new ultracentrifugation under the same conditions. EVs were recovered from the pellet, resuspended in sterile PBS, and stored at 4°C. The procedures follow the Minimal Information for Studies of Extracellular Vesicles 2018 [11].

### 2.5. Scanning Electronic Microscopy (SEM)

Poly L-lysine solution (200 *μ*L) at 0.01% (Sigma-Aldrich), prefiltered through a 0.22 *μ*m pore size filter, was added onto circular coverslips (13 mm, Glasscyto). After 30 min, the poly L-lysine solution was removed, and the wells washed with filtered water and covered with 50 *μ*L containing 1 × 10^6^ trypomastigotes in PBS. After 30 min incubation at room temperature (RT), parasites were fixed (2.5% glutaraldehyde in 0.1 M sodium cacodylate buffer), followed by 1 h incubation (RT). Samples were stored (4°C) prior to electron microscopy scanning at the Federal University of São Paulo facility (CEME, UNIFESP), as previously described [5].

### 2.6. Nanoparticle Tracking Analysis (NTA)

The material released by the parasites was characterized by size and concentration using NTA (NanoSight, NS 300, Malvern, equipped with a CCD camera). Samples were either analyzed pure or diluted (1 : 10) in filtered PBS. Each capture was performed in triplicate always using the same threshold (24.7–24.9°C, 30 sec, camera level set to 10). Results were expressed in concentration (particles/mL), size, and distribution of EVs using NTA software (version 2.3 build 0017).

### 2.7. EV Labeling and Reincorporation in the Parasite

Purified EVs were concentrated by ultracentrifugation (18 h at 100,000 × *g*) and particles (5 × 10^8^/mL incubated with 2 *μ*M PKH26 Red Fluorescent cell dye (MINI26, Sigma-Aldrich). After 30 min at 37°C (dark), the mixture was diluted 5x with PBS and ultracentrifuged (18 h, 100,000 × *g*). The pellet was resuspended with Diluent C, and the number of EVs determined by NTA. Different concentrations of labeled EVs were incubated with trypomastigotes (1 × 10^6^/mL) in DMEM containing 5% glucose. Parasites were collected by centrifugation at different time points (5 min at 1,000 × *g*), washed once with PBS, and resuspended in PBS-0.5% p-formaldehyde. Samples were analyzed by flow cytometry (Fortessa, BD).

### 2.8. Bone Marrow-Derived Macrophage (BMDM) Interaction with T. cruzi EVs

Bone marrow cells obtained from 6- to 8-week-old female C57Bl/6 mice were submitted to differentiation in macrophages by culturing for 7 days in RPMI (Vitrocell Embriolife) culture medium containing 10% FBS, with the addition of L929 supernatant as previously described [12]. 1 × 10^6^ macrophages were plated in 6-well plates. The cells were stimulated for 12 h with 50 ng/mL IFN-*γ* and 500 ng/mL LPS (L2330, Sigma-Aldrich). These preactivated macrophages were then incubated for 24 h with 1 × 10^8^ EVs released by *T. cruzi* from different conditions. The cell culture supernatant was stored at 4°C for analysis by NTA, and the cells were extracted for cytokine analysis.

### 2.9. Gene Expression by qRT-PCR

RNA was extracted from BMDM using RNeasy Plus Microkit (QIAGEN). All samples were also submitted to fluorometry analysis and DNAse treatment. Complementary DNA (cDNA) synthesis was performed using SuperScript II reverse transcriptase kit (Life Technologies), as previously described [13, 14]. Gene expression (qRT-PCR) used SYBR Green-based system detection (Applied Biosystems, Life Technologies). Each reaction was composed of 2 *μ*M of forward and reverse oligonucleotides for each target gene (expression levels were normalized to those of the GAPDH control), 10 *μ*L of the SYBR Green PCR master mix (Applied Biosystems), and 3 *μ*L of cDNA 1 : 2.5 diluted cDNA. Cycling reactions were carried out in the Applied Biosystems 7500 System (Applied Biosystems) starting with one cycle of 50°C (2 min) and 95°C (1 min), followed by 45 cycles at 95°C (15 sec) and 60°C (1 min). Melting curves were determined with an additional cycle of 95°C (15 sec), 60°C (20 sec), and 95°C (15 sec), as previously described [13]. The reference genes using the 2^(-*ΔΔ*Ct)^ cycle threshold method, as previously described [13, 15, 16]. All qRT-PCR procedures were performed following the MIQE guidelines [16]. Each reaction was made in triplicate. The oligonucleotides used for amplification of each target gene are described in Table 1.

### 2.10. Statistical Analysis

Statistical analysis was performed using GraphPad Prism version 6 (GraphPad Software, La Jolla, CA, United States). Data were analyzed using one-way ANOVA test. *p* values < 0.05 were considered significant.

## 3. Results

### 3.1. Kinetics of EV Release under Different Conditions

**Figure 1:**
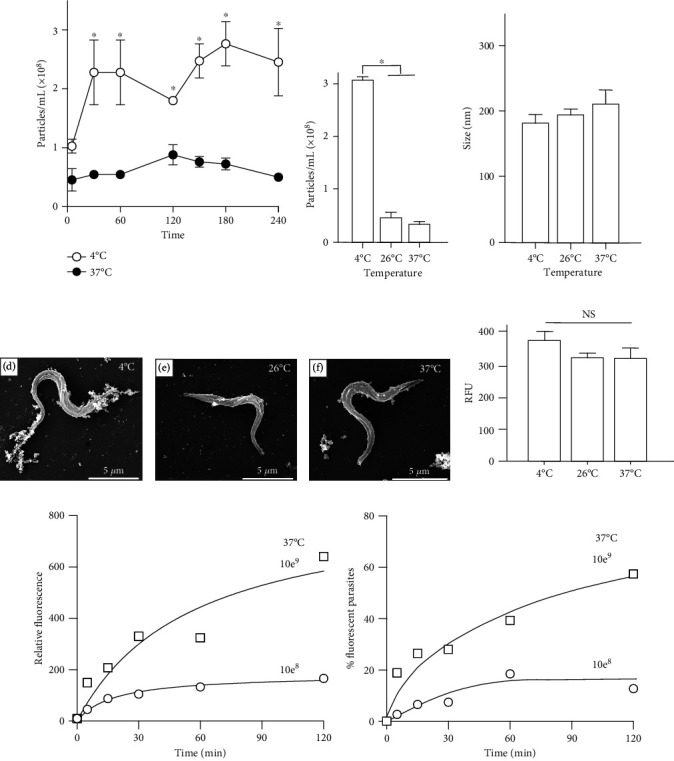
Kinetics of EV release by *T. cruzi* trypomastigote forms. 1 × 10^7^ trypomastigotes were incubated in DMEM supplemented with 5% glucose at different temperatures. EV concentrations were determined at different time points by NTA (see Material and Methods). The panels show total EV concentration (a, b) and particle size (c) in the supernatants after 2 h of incubation at different temperatures (^∗^<0.05). SEM images of trypomastigotes incubated for 2 h at 4°C (d), 26°C (e), and 37°C ((f), size bars = 5 *μ*m). Panel (g) indicates the correspondent relative fluorescence (RFU) of PrestoBlue viability reagent (mean and standard deviation, *n* = 3) showing no significant (NS) differences (*p* > 0.05). EVs labeled with PKH26 (1 × 10^8^/mL and 10^9^/mL) were incubated with 1 × 10^7^ trypomastigotes/mL (37°C). The parasites were collected at different time points, and the median of fluorescence (h) or the percentage of labeled parasites (i) was evaluated by flow cytometry. Results are average values in triplicate measurements.

To follow the process of EV release by trypomastigotes derived from infected mammalian cells, their kinetics under different conditions were evaluated. EVs were collected after 30 minutes of parasite resuspension in the serum-free DMEM containing 5% glucose. The release was higher at 4°C (Figure 1(a)). At 37°C, EVs increased progressively in the supernatant and reached a maximal value at 120 minutes. The number of particles released by the parasites was significantly higher after incubation of trypomastigotes at 4°C compared to 26°C and 37°C (Figure 1(b)), without changes in size (Figure 1(c)). In all cases, the size of EVs was approximately 150 nm, as detected by NTA. SEM also showed a larger amount of material associated with the parasites at 4°C compared to those at 26°C and 37°C (Figures 1(d)–1(f)). No significant differences in the PrestoBlue signal activity, suggesting that the cell metabolic activity was not largely affected in trypomastigotes (Figure 1(g)) that were preincubated for 2 h from 4 to 37°C as shown underneath each image.

To evaluate parasites' ability to release/uptake EVs, they were labeled with PKH26 fluorescent probe, a dye that is incorporated into the glycocalyx of the membranes. Labeled EVs were incubated with trypomastigotes at 4°C or 37°C. A concentration-dependent increase not only in the internal fluorescence but also in the percentage of fluorescent parasites was detected (Figures 1(h) and 1(i)). This confirms that EVs in the medium can be reincorporated.

### 3.2. EV Release Depends on Membrane Integrity

**Figure 2:**
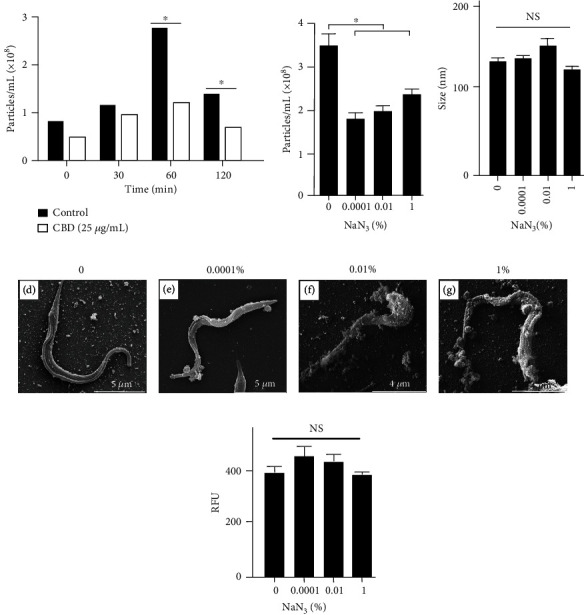
EV release (particles/mL), size (nm), and membrane integrity under chemical stress. *Trypanosoma cruzi* trypomastigotes were incubated in the absence or presence of methyl-beta-cyclodextrin (CBD) (25 *μ*g/mL) or NaN_3_ (0.0001 to 1%) for 2 h at 37°C. EVs in the supernatant were quantified by NTA in triplicate (^∗^*p* < 0.05). The panels show EV concentrations (a, b) and size (nm) (c) in the presence of chemical agents. SEM of trypomastigotes preincubated with the indicated concentrations of NaN_3_ (d–g). Size bars are defined in each image. The relative fluorescence (RFU) of PrestoBlue viability reagent (mean ± standard deviation, *n* = 3) is shown in panel (h).

To test the hypothesis that EV release could depend on membrane integrity [17], parasites were pretreated with *β*-methyl-cyclodextrin (CBD), which affects trypomastigote structure by removing steroids from the membrane surface [18]. At lower concentrations of CBD (0.02 mM), parasites released less EVs than controls (Figure 2(a)). Furthermore, the inhibition was not reestablished up to two hours after treatment, when no morphological changes were detected in the parasites. This suggests that small membrane perturbations affect EV secretion.

### 3.3. EV Discharge from the Parasite Is Dependent on Energy Sources

Since EV release increased at low temperature, the effect of sodium azide, known to partially inhibit *T. cruzi* oxidative phosphorylation and interaction [19, 20], was evaluated. The reductive capacity towards resazurin (PrestoBlue) was detected only at 1% of NaN_3_ (Figures 2(g) and 2(h)). This suggests that even in the presence of azide, *T. cruzi* is still able to perform oxidative phosphorylation through a modified electron transport chain [19]. Sodium azide decreased the number of EVs in the supernatant of the parasite in all concentrations (Figure 2(b)), but no changes in the size was noticed. SEM images, however, showed that the parasite membrane became more granulated at higher NaN_3_ concentrations, and large blebs of parasite membrane are seen attached to the surface (Figures 2(d)–2(g)).

### 3.4. Trypomastigote EV Release Is Boosted in Acidic Conditions

**Figure 3:**
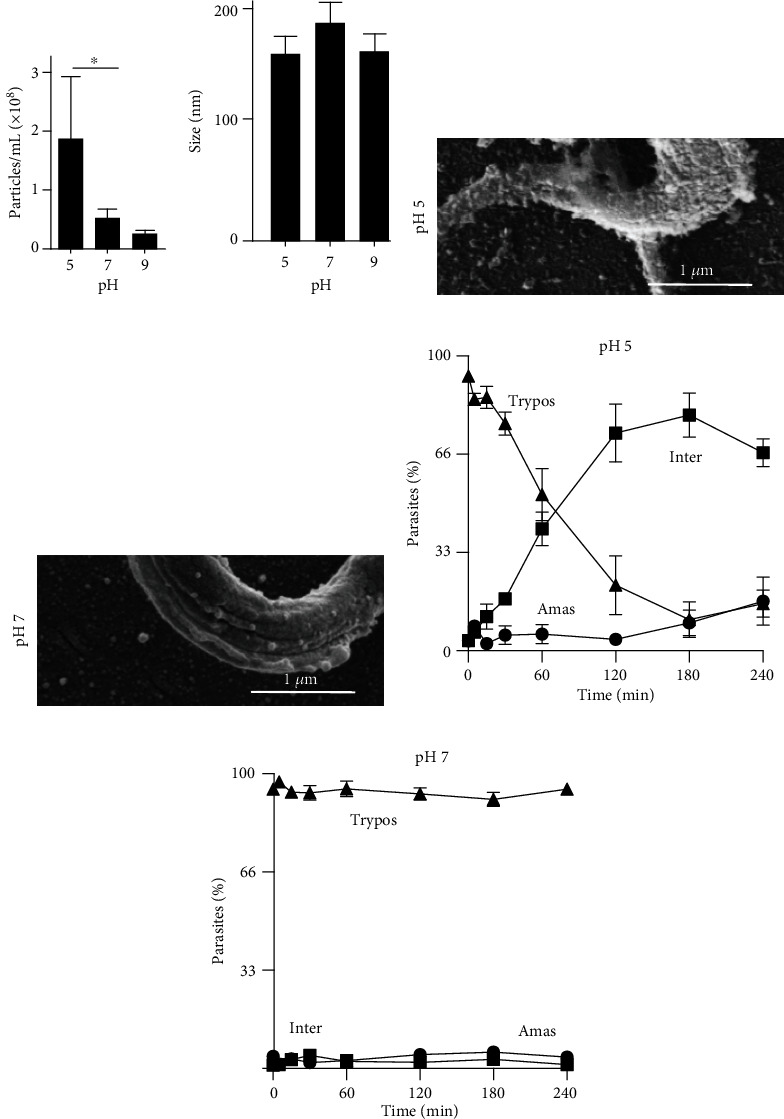
pH effect on EV release. Trypomastigote (1 × 10^7^/mL) forms incubated (2 h, 37°C) in culture medium containing 5% glucose previously adjusted to the indicated pH values. EV concentrations (a) and sizes (b) were analyzed by NTA (^∗^*p* < 0.05). After incubation at pH 5 (c) or pH 7 (d), parasites were analyzed by SEM. In parallel, parasites were incubated at pH 5 (e) or pH 7 (f), stained with Giemsa, and 300 parasites were evaluated according to their form: trypomastigotes (Trypos), intermediate (Inter), or amastigote (Amas). Their percentages were quantified in triplicate.

After mammalian cell invasion, the parasite faces pH changes. Therefore, we examined the release of EVs after 2 h of incubation at pH 5, 7, and 9. The parasite did not have significant changes in cell metabolic activity at pH 5, a situation found inside cell lysosomes after cell invasion [21], while some decrease occurred at pH 9. The number of secreted EVs largely increased at pH 5 compared to pH 7 and pH 9 (Figure 3(a)), and there was no variation in the size of EVs (Figure 3(b)). This increased release can be seen by SEM images when trypomastigotes incubated under acidic conditions had more particles covering their surface compared to neutral conditions (Figures 3(c) and 3(d)). As already reported [22], at pH 5, trypomastigotes change shape and after 2 hours, they start to differentiate into amastigotes (Figure 3(e)). At pH 7, they remain unchanged (Figure 3(f)).

### 3.5. Effect of Nitrosative Stress in the EV Release

**Figure 4:**
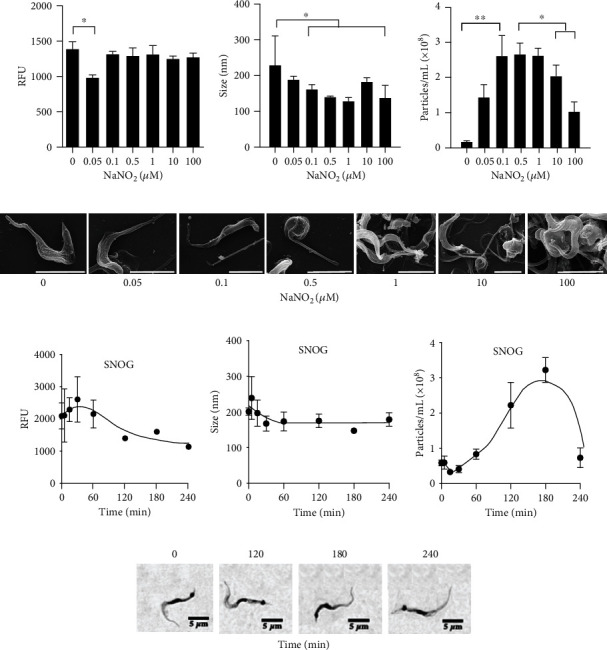
Effect of nitrosative stress on EV release by *T. cruzi* trypomastigotes. 1 × 10^7^ trypomastigotes were incubated for 2 h at 37°C in culture medium adjusted to pH 5.0 with different concentrations of NaNO_2,_ including the control with the salt. After the incubation period, cell viability was evaluated by the PrestoBlue assay(a) and EV size (nm) (b) and EV concentration (particles/mL) (c) were measured by NTA (^∗^*p* < 0.05 and ^∗∗^*p* < 0.01). SEM of trypomastigotes releasing EVs incubated under different NaNO_2_ concentrations (d). Size bars = 5 *μ*m. Alternatively, trypomastigotes were incubated at 37°C in DMEM supplemented with 5% glucose containing 100 *μ*M SNOG. Parasite viability was evaluated at the indicated intervals by PrestoBlue assays (e), and the size (f) and concentration (g) of EVs in the supernatants were determined by NTA (*n* = 3). Panel (h) shows pictures of parasites stained by Giemsa after each incubation period.

The major defense that eliminates *T. cruzi* in the mammalian host is nitrosative stress caused by nitric oxide (NO) [23]. We evaluated the effect of distinct NaNO_2_ concentrations, from 0 to 100 *μ*M, at pH 5.0 on EV production. At this pH, NaNO_2_ is soluble and spontaneously produces NO [24–27]. We observed a small effect in cell viability only at the concentration of 0.05 *μ*M (Figure 4(a)). However, a major decrease in the size of EVs (Figure 4(b)) along with an increase in NaNO_2_ was noticed (Figure 4(c)). SEM images showed that parasite integrity was conserved in all groups (Figure 4(d)).

The EV release induced by NO was confirmed by using S-nitrosoglutathione (SNOG), a NO generator that can be used at neutral pH. Trypomastigotes were incubated in DMEM with SNOG (100 *μ*M), a toxic condition for cells. A decreased metabolic activity was detected (Figure 4(e)). In parallel, parasites released more EVs, reaching a maximum at 180 minutes without significant changes in size (Figures 4(f) and 4(g)) or morphology (Figure 4(h)). These results indicated that EV release by trypomastigotes is increased under nitrosative stress.

### 3.6. Immunomodulatory Role of EVs Released under Different Stress Conditions in BMDM

**Figure 5:**
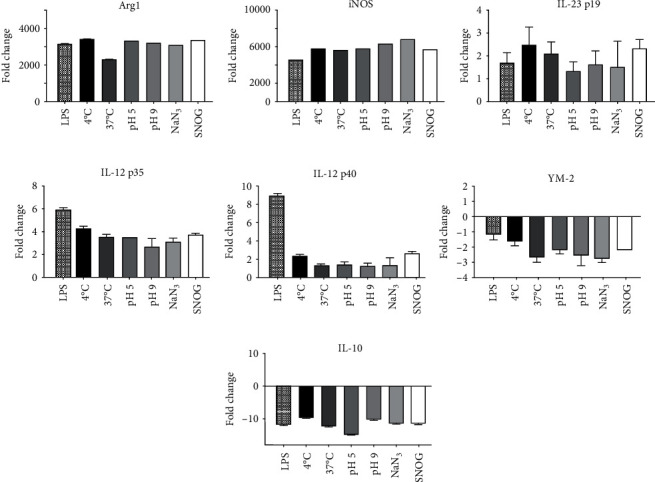
Immunomodulation of EVs obtained from *T. cruzi* submitted to different stress conditions. IFN-*γ*-primed macrophages (BMDM) were incubated (24 h, 37°C) with *T. cruzi* EVs. LPS from *E. coli* was used as positive control. qRT-PCR was performed to detect the expression of immune response genes: Arg 1 (a), iNOS (b), IL-23 p19 (c), IL-12 p35 (d), IL-12 p40 (e), YM-2 (f), and IL-10 (g) (*n* = 3, ^∗^*p* < 0.05).

To investigate the immunomodulatory functions of EVs isolated under different stress conditions, IFN-*γ*-treated BMDM were exposed to them; differential gene expression was determined. In general, EVs activated BMDM in a similar manner of LPS, except for IL-12-p40 (Figure 5). No major differences were observed among the different types of EVs, suggesting that their functional cargo properties had a negligible effect under stress conditions.

## 4. Discussion

In nature, parasites deal with numerous environmental changes in vertebrate and invertebrate hosts, including variations in temperature, pH, and reactive oxygen and nitrogen species. In fact, these factors modulate signaling cascades that regulate parasite infection, proliferation, and survival [28]. Previous studies have already shown that different stages of *T. cruzi* release EVs under physiological conditions. They modulate NO and cytokine production by macrophages and delay parasite migration in the gut of vector [29, 30]. Here, several experiments evaluated the role of stress factors affecting EV release by *T. cruzi* trypomastigotes.

Studies on how temperature affects EV release in pathogens are very scarce. Early studies have already shown for *Leishmania mexicana* promastigotes, another trypanosomatid, that EV release increased at 37°C, but not at 4°C [31]. Interestingly, for *T. cruzi* trypomastigotes, we found that larger amounts of EVs accumulate at low temperature (4°C). Changes on the parasite surface organization, known to form patches containing different sets of components in the membrane, may be involved in this process in response to different environments [17]. Such distribution may change with temperature variations. For example, *Leishmania* promastigotes occur in the vector, where temperatures are around 25-26°C, whereas *T. cruzi* trypomastigotes are mainly found in the mammalian host (37°C). Most of the trypomastigote surface is composed of glycosylphosphatidylinositol- (GPI-) anchored proteins [32], and the lipid moieties are largely affected by decreasing the temperature. In fact, changes in temperature are largely sensed by *T. cruzi* triggering changes in gene expression [33]. It is also possible that the accumulation was due to diminished reincorporation at low temperatures. However, this possibility is unlikely as the difference in the release at 4°C was rapid, much faster than the kinetics of reincorporation at 37°C. Consistent with these observations, we observed that trypomastigote EVs are rapidly released and reincorporated. This may explain why after longer incubation periods, the amount of EVs in the supernatant reaches a saturation point. Uptake could be related to interparasite signaling as observed for *T. brucei* [34, 35]. It secretes EVs as nanotubes that are also released in knockouts of the Vps36. This protein is part of the endosomal sorting complexes required for transport (ESCRT) involved in exosome formation. In *T. cruzi* metacyclic trypomastigotes, a stage that corresponds to infective forms generated in the insect vector, EVs confer serum complement resistance in susceptible parasites [9], but in general, paracrine signaling is poorly related in *T. cruzi*. Therefore, more studies are required to evaluate whether EV reincorporation has a role for mammalian stages of *T. cruzi*. Since temperature affects EV secretion, our next step was to evaluate if chemical agents could also modulate this phenomenon in *T. cruzi* trypomastigotes.

Our results showed that methyl-*β*-cyclodextrin (CBD) inhibited EV secretion in a concentration that maintains parasite integrity. This supports the idea that the process depends on the membrane bilayer organization. CBD removes sterol, required for the assembly of lipid domains, from membranes [36]. These domains are observed in trypomastigotes, and the presence of lipid chains and cholesterol or ergosterol boundaries might be involved in EV release. In fact, two different EV populations enriched in mucins and members of TS family of glycoproteins are detected [18]. CBD prevents Ca2+-dependent release of EVs from platelets [37] and other cells [38]. Further experiments are required to evaluate whether divalent cations affect EV release and the dependency of the negative charges provided by parasite sialylation. Azide inhibits trypomastigote adhesion to mammalian cells, most likely by preventing lateral diffusion of membrane components [20]. Like CBD, sodium azide also decreased EV production, although no major changes in parasite's viability were detected. Interestingly, at higher concentrations, the parasite surface became more granular, covered with several larger vesicles that remained attached to the membrane. In the same manner, it inhibits EV release. In summary, CBD and azide affected EV release by trypomastigotes without a negligible effect in the size. At higher concentrations, visible effects were detected in the parasite surface morphology.

During their life cycle, trypomastigotes enter mammalian cells, differentiate into amastigotes in parasitophorous vacuole, and can be retained in the cell, which is consistent with both the data in the literature and our own [39]. This is also a common described phenomenon [22, 40] when parasites grow for longer periods in acidified culture medium. Interestingly, here, EV release was increased under acidic conditions and the surface became granular, suggesting significant alterations in membrane. However, like azide, no major changes in the size were detected. Consistent with the literature, at pH 5, trypomastigotes start to differentiate into amastigote-like forms. Under acidic conditions, epimastigotes, the forms found in the vector, release membrane lipids [41]. *Trypanosoma cruzi* epimastigotes exhibit changes in morphology, especially in epimastigotes [42], which after 24 h induce large metabolic alterations [43]. However, it is still unclear how acidic conditions induce parasite changes over short periods. The EV release may be increased by alterations in the subpellicular cytoskeleton interaction with the membrane at pH 5 or through the activation of an acid-dependent and o-phenanthroline-insensitive GPI-phospholipase present in trypomastigotes [44]. Interestingly, vesicles released by epimastigotes are enriched in phospholipids compared to the cell, but no increase in lysophospholipids was observed [45]. In a preliminary analysis, in trypomastigote EVs, we detected the presence of phosphatidylethanolamine and phosphatidylserine, absent in epimastigotes, by thin-layer chromatography and gas chromatography followed by MALDI spectrometry analysis (in preparation).

Finally, we decided to focus on nitrosative stress, since this is also a common environment faced by the parasites inside the cells. We also noticed that the release of EVs increased with NO-produced NaNO_2_ during medium acidification. A similar increase was observed at pH 7 by using SNOG as a NO source. These effects are relevant, because the interaction of trypomastigotes and macrophages triggers the activation of the innate host immune response, characterized by the production of proinflammatory cytokines, activation of the NADPH oxidase complex, and the inducible nitric oxide synthase (iNOS) [46]. These enzymes produce radical superoxide (O_2_^−^) and NO, respectively [47]. These are responsible for the elimination of the parasite due to its trypanocide effect [48]. It is possible that the observed increase may be associated with the parasite's response to the stress suffered, a reflection of the mechanisms triggered by the protozoan to resist this stress, for example, via the trypanothione-dependent antioxidant system. Those events may be related to several factors, as a mechanism employed by the parasite to resist and survive nitrosative stress, involving intracellular signaling cascades that directly interfere with vesicular biogenesis. SNOG, as other NO donor molecules, is capable of inhibiting *T. cruzi* cruzipain (CZ), a cysteine protease, *L. infantum* cysteine proteinase, and *P. falciparum* falcipain by *S*-nitrosylation of the catalytic residue of the enzymes [49, 50]. These observations might, therefore, suggest a role for this major surface protease in the trypomastigotes in EV release.

*Trypanosoma cruzi* EVs display high proinflammatory profile depending on the strain [51]. Consistent with these observations, here, the EVs increased the expression of iNOS, IL-12, and IL-23 genes, typical of M1-like phenotype, as previously observed [29]. EVs from *T. cruzi* contain a wide range of pathogen-associated molecular patterns (PAMPs) that contribute to their ability to interact with the cellular milieu in the vertebrate hosts. Those include GPI-mucins, *trans*-sialidases, and cruzipains [3]. Here, regardless of the stress conditions applied to the trypomastigotes, the functional properties of EVs were not affected. This suggests that stress conditions are more likely to affect quantity rather than quality of the EVs.

## 5. Conclusion

In conclusion, the release of *T. cruzi* EVs is dependent on factors found in the variable environmental conditions faced by the parasite. As the EVs confer increased infectivity and virulence in a mammalian host [2], the stress-induced release is clearly a response to environmental changes. We can associate the release with a mechanism of surface budding dependent on the membrane organization. This is based on the variable size of EVs, their composition like the plasma membrane, and the rapid release upon stress. It is still unknown whether the release occurs in a similar way in host tissues or in the blood and what key molecular events are taking place during the vesiculation and release from the parasite surface. This work provides an initial support to answer these relevant questions, to get a better understanding of Chagas disease and help to deal with it.

## Figures and Tables

**Table 1:** Oligonucleotides used for amplification, sequence, and gene of each target gene.

Oligonucleotide	Sequence	Gene
Arg1Fow	5′-GAGACAGGGAAGTCTGAAGCAC	Arginase 1
Arg1Rev	5′-CATTGGCTTGCGAGACGTAGAC	Arginase 1
iNOS Fow	5′-ATGGACCAGTATAAGGCAAGC	iNOS
iNOS Rev	5′-GCTCTGGATGAGCCTATATTG	iNOS
IL-23p19 Fow	5′ AATAATGTGCCCCGTATCCAG	IL–23p19
IL-23p19Rev	5′ GCTCCCCTTTGAAGATGTCAG	IL–23p19
IL-12p35 Rev	5′-ACGAGAGTTGCCTGGCTACTA	IL-12p35
IL-12p35Fow	5′-CCTCATAGATGCTACCAAGGCAC	IL-12p35
IL-12p40 Fow	5′-TTGAACTGGCGTTGGAAGCACG	IL–12p40
IL-12p40 Rev	5′-CCACCTGTGAGTTCTTCAAAGGC	IL–12p40
Ym2Fow	5′-GTGACCCTACTGTTAGTGCTGG	YM2
Ym2Rev	5′CGGGAAGACAATAACTGCACCC	YM2
IL-10 Fow	5′GACTTTAAGGGTTACCTGGGTTG	IL-10
IL-10 Rev	5′TCACATGCGCCTTGATGTCTG	IL-10
GAPDH forward	5′AAATGGTGAAGGTCGGTGTG	GAPDH
GAPDH reverse	5′TGAGGGGTCGTTGATGG	GAPDH

## Data Availability

All data used to support the finding of this study are available from the corresponding authors upon request.
